# Pharmacotherapy in Tobacco Cessation: A Narrative Review

**DOI:** 10.7759/cureus.35086

**Published:** 2023-02-16

**Authors:** Devanjali D Pajai, Priyanka Paul, Amit Reche

**Affiliations:** 1 Public Health Dentistry, Sharad Pawar Dental College and Hospital, Datta Meghe Institute of Higher Education and Research, Wardha, IND

**Keywords:** and nicvax, naltrexone, anxiolytics, nortriptyline, clonidine, mecamylamine, varenicline, bupropion

## Abstract

A chronic, recurring illness, known as nicotine addiction and dependence, is defined by a person's dependence on the substance up to the extent that their normal day-to-day activities are compromised in the absence of the substance. This paper will highlight first-line smoking cessation treatments, such as nicotine replacement therapy (NRT), bupropion, and varenicline, and second-line medications, such as clonidine, nortriptyline, anxiolytics, mecamylamine, naltrexone, and NicVAX (Nabi Biopharmaceuticals, Rockville, MD, USA). NRT offers many options for nicotine delivery methods, comprising nicotine gum, rapid-release gum, lozenges, transdermal patches, high-dose nicotine patches, oral inhalers, nasal sprays, electronic nicotine delivery systems (ENDS), and sublingual tablets. Pharmacotherapies for quitting tobacco should lessen withdrawal symptoms and stop nicotine's reinforcing effects without having too many side effects.

## Introduction and background

Smoking is one of the world's most common causes of death regardless of the accumulating evidence of health risks of tobacco products that have gathered over the last 70 years. The global adult smoking prevalence in 2020 was 6.5% (6.3%-6.7%) for females and 32.6% (32.2%-33.1%) for males. A total of 1.18 billion (0.94-1.47) individuals routinely consumed tobacco in 2020, causing 7 million (2-11.2) deaths [1]. Worldwide, both male and female adolescents used tobacco at high rates. The research that is now available points to a clear link between smoking and poor dental health in teenagers. Gingivitis (72.8%), gingival bleeding (51.2%), and oral malodor or halitosis (39.6%) are all common in teenagers who smoke frequently. Teenagers who smoke frequently have a higher risk of developing hyperkeratosis, smoking-related melanosis, hairy tongue, and dental caries [2].

Nicotine dependency requires medical care, just as any other substance use condition or chronic illness. The patient should be informed about the advantages of quitting, the method, as well as any potential withdrawal symptoms. A treatment plan that works for the patient should be selected with their cooperation [3]. The most significant pharmacological agent is nicotine, a potentially deadly alkaloid (1-methyl-2-[3-pyrodyl] pyrrolidine) that affects pathologic bodily functions. Nicotine in tobacco smoke causes tolerance to its effects over time, in addition to altering the pathophysiology of smokers' bodies. One strategy for increasing life expectancy and lowering morbidity is to stop smoking [4]. By warning patients about the risks of tobacco use immediately as the oral cavity shows the first signs of tobacco use, dentists play a crucial part in reducing the high death rates brought on by tobacco use. As it doubles the success rate of quitting smoking across all intervention levels, the most extensive support increases the effectiveness of nicotine replacement therapy (NRT) [5].

A whopping 50% of chronic cigarette smokers die too soon from diseases, including cancer, heart disease, lung disease, or other ailments caused by smoking [6]. Quitting smoking can significantly lower this risk, and the earlier in one's smoking career that it happens, the better. As the chemical nicotine is very addicting, the vast majority of persons who smoke frequently are dependent on tobacco usage rather than just being addicted to nicotine. A reliance forms when regular stimulation and the easing of withdrawal symptoms are temporally related to sensory inputs and rituals. Systems for NRT gradually release nicotine and do not raise plasma nicotine levels to lower the risk of addiction because of this important association. In conjunction with minimizing or eliminating withdrawal symptoms, smoking cessation medications should work to lessen nicotine's positive reinforcing effects. Furthermore, nicotine addiction-related receptor subtypes should be the target of pharmacotherapy, while receptors that may produce unwanted side effects should be avoided [7].

Given the high mortality rate and prevalence of tobacco usage, this review summarizes the studies on the many types of NRT now used to treat nicotine dependence as well as other medical treatments for tobacco use disorder.

## Review

Methodology

**Figure 1:**
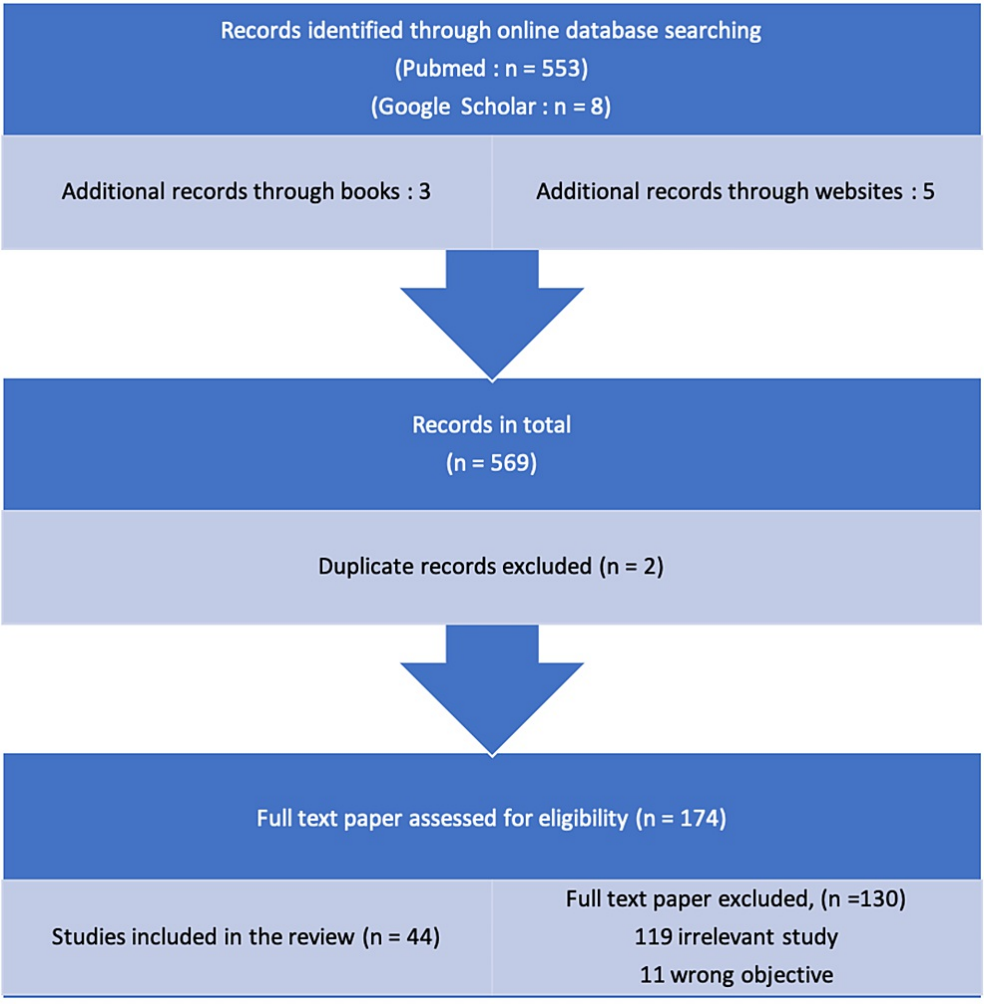
Flowchart showing the selection of included research studies. Figure credits: Devanjali D. Pajai.

The objectives of this review are as follows: Pharmacotherapy for tobacco cessation. Using the online databases PubMed, Google Scholar, and Medline, a search of the English-language literature was conducted. The search keywords were pharmacotherapy in smoking cessation, nicotine gum, rapid-release gum, nicotine lozenges, transdermal patch, high-dose nicotine patches, nicotine sublingual tablet, nicotine oral inhaler, nicotine nasal spray, electronic nicotine delivery systems (ENDS), NRT, bupropion, varenicline, clonidine, nortriptyline, anxiolytics, naltrexone, mecamylamine, NicVAX (Nabi Biopharmaceuticals, Rockville, MD, USA). The author aimed to cover all the recent and existing pharmacological approaches that could help treat addiction. Studies in English, research within the past 10 years, and studies only focused on pharmacological treatments for tobacco use disorder all meet the criteria for inclusion in this review. In total, 44 articles were included in this review (Figure 1).

NRT

It has been established that providing nicotine can minimize nicotine and tobacco withdrawal symptoms as well as smokers’ desire to smoke in the days and weeks after quitting [4]. Now that NRT has gained widespread acceptance as a technique for helping smokers stop, several clinical recommendations suggest utilizing NRT as the first treatment choice for smokers seeking pharmacological assistance [5]. Depending on the country, NRT is either marketed as a transdermal patch or as a buccal absorption device (gum, lozenge, nasal spray, inhaler, or sublingual pill) [4].

Nicotine Gum

The first NRT product that was readily available was nicotine gum (Nicotine Polacrilex, McNeil Consumer Healthcare, Fort Washington, PA, USA). It is offered in doses of 2 and 4 mg. According to studies, 4 mg of chewable gum has a higher success rate than 2 mg in helping smokers quit smoking [6]. There was no distinction between starting the nicotine gum medicine four weeks earlier or starting it on the designated quit date [7]. Neuronal nicotinic acetylcholine receptors are present in the brain. However, this does not mean that intakes and effects are always consistent. This could be based on the argument that different people consume different amounts of nicotine gum, depending on their daily intake limits in addition to other variables such as age, body mass index, gene expression, peak concentration, and age [8].

Rapid-Release Gum

The gum used in rapid-release products has a remarkable gum base that enables both biphasic nicotine delivery and speedy initial nicotine release. Furthermore, it elevates the pH to hasten absorption through the oral mucosa [9]. Compared to regular nicotine gum, rapid-release nicotine gum offers an advantage because it is quick and completely relieves nicotine cravings, according to a study that compared the effectiveness of Nicorette (McNeil Consumer Healthcare) and rapid-release nicotine gum in reducing cravings brought on by smoking cues [6].

Nicotine Lozenges

Nicotine lozenges can be used in place of nicotine gum by patients who need sporadic and periodic nicotine dosages but are not able to chew gum for an extended amount of time. They are available in doses of 1, 2, and 4 mg [10]. Although there was a noticeable decrease in *desire to use tobacco* (i.e., craving) compared to baseline during the first two weeks of quitting, users of smokeless tobacco who took nicotine lozenges did not notice any differences in their overall tobacco withdrawal symptoms. Smokeless tobacco users tolerated and approved of the nicotine lozenge [11].

Transdermal Patch

Nicotine is progressively absorbed through the skin once nicotine patches are applied [12]. Smokers with a greater dependence level can use the strongest patches, while those with a lower dependence level can use a lesser dosage. Thanks to the range of dosages, users can progressively reduce their nicotine intake over a few weeks or longer, letting their bodies acclimatize to reduced nicotine doses and eventually reach a nicotine-free state [5]. The key advantage of nicotine patches over acute NRT formulations is the simplicity of compliance; rather than actively using a medicine throughout the day, the patient only needs to apply the patch to their skin in the morning [13]. Localized skin responses are the side effects that are most frequently reported. Skin responses can be minimized by changing the patch application site, as directed, each day [5].

High-Dose Nicotine Patches

Standard 22-mg patches can only restore around half of a smoker's baseline blood nicotine and cotinine levels. As a result, 42 mg of greater transdermal nicotine dosages were investigated. In terms of efficacy, high-dose transdermal NRT achieved a statistically greater abstinence rate [5]. According to the medical literature, high-dose transdermal NRT has not been demonstrated to be safe or helpful for smoking cessation [14].

Nicotine Sublingual Tablet

Because the pill is taken sublingually, there is no need to chew it. It is recommended that it should be used for at least 8 to 12 weeks before progressively reducing the number of tablets [12]. For people who are very dependent on nicotine, 16 to 24 sublingual tablets per day (i.e., a maximum of 302 mg tablets dispersed throughout the day) are recommended, whereas 8 to 12 tablets per day are recommended for those who have a low reliance [15]. It should be taken with caution in persons who are addicted to nicotine. The two most common side effects are mouth soreness and sleeplessness [16].

Nicotine Oral Inhaler

A brand-new inhaler that mimics many of the smoking rituals while administering nicotine aerosol has been developed. The device is similar to a regular cigarette in size and form, and it features a tiny breath-operated valve that allows the user to control how much air they inhale. Because of this, the amount of *puffs* in one charge (or *dose*) of the inhaler device that is regulated by the user's depth of inhalation determines the speed at which nicotine is administered from that charge [17]. A nicotine inhaler typically resembles a cigarette or cigar and is composed of a mouthpiece and a plastic cartridge filled with nicotine. Ten milligrams of nicotine are contained in each cartridge [18]. The mouth, esophagus, and stomach deliver about 36% of the nicotine, while the lungs only receive 4% of it. Because the inhaler absorbs nicotine at the exact pace as nicotine gum, caution is required to avoid placing it on the lips [19].

Nicotine Nasal Spray

When compared to a placebo, nasal spray nearly doubles the quit rate [20]. Nicotine patches and nasal sprays reduce fetal nicotine exposure when compared to smoking. The fastest mode of delivery, nasal spray, most closely resembles the rise in nicotine levels observed when smoking. Because it quickly reduces appetite, the nasal spray is most beneficial to highly dependent smokers [21].

ENDS

The effect of cigarettes on quitting smoking continues to remain up for argument. Recent systematic studies and meta-analyses found a 28% lower chance of smoking cessation among e-cigarette users. Some pros and cons of ENDS are mentioned later.

Pros: The experience was comparable to that of a regular cigarette for the customer. In addition to offering a variety of tasty flavors and appealing designs, ENDS are thought to be less dangerous than regular cigarettes. They have lower concentrations of tobacco-specific nitrosamine and volatile chemical compounds than in conventional cigarettes [22].

Cons: E-cigarettes are frequently the first nicotine-containing product used by young people. ENDS may also serve as a starting point for using other nicotine products, such as regular cigarettes. Several e-liquids contained aldehydes and ketones, metals, additional tobacco-specific nitrosamines, and volatile organic chemicals in a wide range of concentrations. The prevalence of serious lung illnesses such as chronic obstructive pulmonary disease (COPD), asthma, and lung cancer are linked to vaping and has been rising rapidly [23].

Bupropion

Although this medication is an antidepressant, it is unknown how it works to treat nicotine addiction [22]. Whether a smoker is depressed or not, bupropion with continuous release is an excellent aid in helping them quit. It is equally effective as the nicotine patch and monotherapy [24]. One to two weeks before the patient's anticipated termination date, a dose of 150 mg of bupropion is typically started. The dosage must be increased after three days to 150 mg twice daily for 7 to 12 weeks. As long as abstinence is kept up, bupropion can be administered for up to 12 months (maintenance dose = 300 mg/day) [25]. One of the adverse effects is insomnia, which is less common if the medication is given at least eight hours before going to bed. Other side effects include headaches, dizziness, diaphoresis, weight loss, xerostomia, nausea, and vomiting [26]. People who are affected by seizures should not use bupropion; as bupropion can lower the seizure threshold, it is also contraindicated in severe diseases such as brain intravascular malformation, major head injuries, stroke, tumors, or infection of the CNS. Anorexia or bulimia; sudden alcohol withdrawal; current use of benzodiazepines, barbiturates, or antiepileptic medications; and the use of linezolid or intravenous (IV) methylene blue, both of which contain reversible monoamine oxidase inhibitors, together with recently using monoamine oxidase inhibitors, are all contraindications [24].

Varenicline

Varenicline is a nicotinic acetylcholine receptor partial agonist [27]. Dopamine, the principal neurotransmitter associated with nicotine addiction, can be released through these receptors. Partially agonist action alleviates withdrawal symptoms [25]. Titration starts with 0.5 mg administered orally once daily for three days and then increased to 1 mg given orally twice daily for the final three days of the course of treatment. In patients who have unfavorable side effects, lower dosages of varenicline (such as 0.5 mg twice daily) may be used. Varenicline may be used for up to six months of nonstop abstinence if well tolerated [28]. Frequent adverse effects of using varenicline include nausea, sleeplessness, strange nightmares, headaches, nasopharyngitis, and xerostomia [29]. Patients who have previously experienced significant skin reactions and hypersensitivity responses to varenicline should not use it. Seizure disease is not a contraindication, even when the presence of seizures during therapy necessitates halting the medication [24].

Clonidine

Clonidine, a medicine that acts on the central nervous system, was initially recommended as an antihypertensive to alleviate the withdrawal symptoms from some addictive behaviors. The existing studies suggest that the dose be titrated up to a maximum of roughly 400 g/day, as tolerated, starting with 100 g twice daily (orally or with an equivalent transdermal patch). If clonidine therapy is anticipated before the quit date, it should begin 48 to 72 hours beforehand [30]. In a trial conducted by Hilleman et al., no more smokers dropped out of the study when clonidine was administered in place of a placebo [31]. At all follow-up visits, women on clonidine had a significantly greater rate of abstinence than men. Therefore, only female smokers may benefit from clonidine [32].

Nortriptyline

Tricyclic antidepressant nortriptyline has often shown promise as a tool for quitting smoking [33]. Tricyclics (secondary amines) are a class of drugs that include nortriptyline. These drugs are more frequently referred to as tricyclic antidepressants (TCAs). According to widespread consensus, nortriptyline increases the levels of serotonin and norepinephrine in the synapse by preventing their reabsorption by the presynaptic neuronal membrane. Typically, nortriptyline is consumed orally as a pill or oral solution. The strengths of the capsule form are 10, 25, 50, and 75 mg. Typically, the oral solution version contains the following ingredients: 10 mg/5 mL (473 mL). Adults typically take 25 mg three or four times per day; the dosage should start low and be increased as necessary. The total daily dose may also be administered once daily as an alternative regimen. When nortriptyline is used in doses larger than 100 mg/day, plasma levels should be tracked. A dose of more than 150 mg/day is not recommended [34]. A combined sample of smokers who received a variety of dosages exhibited considerable variation in plasma nortriptyline concentrations, according to Mooney et al. [34]. Only nortriptyline levels that also decreased locomotor activity improved somatic withdrawal symptoms [35].

Anxiolytics

As a form of therapy, anxiolytics have also been suggested. Meprobamate, ondansetron, doxepin, buspirone, diazepam, and the beta-blockers metoprolol, oxprenolol, and propranolol are some of the medications that can help with anxiety, a sign of nicotine withdrawal. For students to successfully quit smoking, this study suggests effective anxiety therapy through psychiatric/psychotherapeutic intervention [36].

Buspirone 

Buspirone is a nonbenzodiazepine anxiolytic that targets serotonin neurotransmitters. It has no sedative or addictive properties. In smoking cessation trials, the maximum daily doses have varied from 30 to 60 mg for 9 to 13 weeks, with therapy starting two to three weeks before the quit date [36].

Diazepam 

In one long-term tobacco cessation research, diazepam was compared to a placebo and clonidine in a randomized fashion by Hao in 1988 in China. It was recommended to take 7.5 to 15 mg daily for four weeks. Additionally, subjects got three private appointments with a psychiatrist [36].

Meprobamate

This sedative was utilized in one long-term smoking cessation study. Meprobamate (400 mg/day) was evaluated by Schwartz in 1968 in a factorial design trial that randomly assigned patients to receive the medication or a placebo, either alone or in conjunction with a group or one-on-one counseling [36].

Ondansetron

Serotonin 5-hydroxytryptamine 3 (5-HT3) receptor antagonists have been recommended for smoking cessation on the theory that they should lessen nicotine's reinforcing effect. 

Beta-Blockers

In a double-dummy experiment with a 12-month follow-up, metoprolol (100 mg twice daily) and oxprenolol (80 mg twice daily) were assessed in 1984 and compared to a placebo [36].

Mecamylamine

To neutralize nicotine's effectiveness in quitting tobacco, it can be taken as an antagonist. The results that follow are quite noteworthy. In a dose-dependent manner, mecamylamine pretreatment decreases the ability of both humans and animals to differentiate between nicotine and placebo. Pretreatment with mecamylamine makes people desire more cigarette smoke when persons are tested using a device that combines the smoke from low- and high-nicotine cigarettes (presumably by lowering its nicotine effects). Mecamylamine pretreatment enhances many indices of tobacco consumption and tobacco smoke consumption when individuals are permitted to smoke [3]. Mecamylamine pretreatment decreases the reinforcer effect of IV nicotine delivery in animals [37] and maybe in humans [38]. When combined with counseling, mecamylamine reduces the desire to smoke in heavy cigarette smokers, and after two weeks of treatment, 50% of patients effectively stop smoking. When mecamylamine use was discontinued, the study's average daily dose was 26.7 mg [39].

Naltrexone

As an opioid antagonist, it has a protracted half-life. The primary goal of treatment for alcohol and opioid use disorders is to change the factors that reinforce euphoric induction. It has a lengthy half-life as an opioid antagonist because it inhibits the mu-opioid receptor. Pharmacologically, naltrexone and its active metabolite, 6-beta-naltrexone, are effective against alcohol and opioids. If there are no withdrawal symptoms after taking a 25 mg oral dose, repeat it after an hour. Naltrexone is available as a 50 mg oral tablet. Naltrexone can be taken orally with or without food. Adverse gastrointestinal (GI) symptoms may be reduced by administration with or after meals. Another kind of naltrexone is a depot injection (380 mg). The top outer quadrant of the gluteal area must be injected using the given needles when administering the Intramuscular version; doctors should avoid injecting into the blood vessel. Drug administration by IV or subcutaneously or into fatty tissue is not advised. Before beginning naltrexone, the patient must undergo an opioid detox [40]. As nicotine may have opioid-mediated effects on performance enhancement and other positive outcomes, the use of naltrexone to quit tobacco is supported [41]. According to David et al. [42], naltrexone does not have any favorable effects on either short- or long-term smoking cessation when used alone or as an adjuvant to NRT.

NicVAX

The adaptive immunity against the nicotine molecule that underlies nicotine vaccination can potentially be a new method of treating tobacco dependence and preventing relapse. This is accomplished by stimulating the immune system to make antibodies that attach to nicotine molecules and expand them, preventing them from crossing the blood-brain barrier and connecting to nicotine receptors, so initiating the pleasurable experience that leads to addiction in smokers [43]. When paired with varenicline and psychological assistance, the nicotine vaccination NicVAX does not seem to increase the likelihood of discontinuing tobacco use [44].

## Conclusions

Pharmacotherapy can help smokers quit. The most effective first-line treatments are NRT and bupropion. NRT works by progressively reducing the nicotine intake by the patient, which eventually ends in habit cessation. Other drugs mainly reduce the withdrawal symptoms by acting as antidepressants or antianxiety, whereas NicVAX works by activating the immune system against nicotine. We still need further research for the new drugs as they have not been approved for routine use but they do seem very promising.
